# Effect of the Addition of Different Amounts of Aramid Fibers on Metal Friction and Wear during Mixing

**DOI:** 10.3390/polym14142961

**Published:** 2022-07-21

**Authors:** Deshang Han, Quyang Ma, Jie Wang, Hongbo Chen, Chuansheng Wang, Wenwen Han

**Affiliations:** 1 College of Electromechanical Engineering, Qingdao University of Science and Technology, Qingdao 266061, China; handeshang@163.com (D.H.); chenhb@qust.edu.cn (H.C.); qustwcs@163.com (C.W.); 2 School of Automotive Engineering, Guangdong Polytechnic of Science and Technology, Zhuhai 519000, China; maquyang15@163.com; 3 Ural Institute, North China University of Water Resources and Electric Power, Zhengzhou 450046, China; wangjie@ncwu.edu.cn; 4 National Engineering Laboratory for Advanced Tire Equipment and Key Materials, Qingdao 266061, China

**Keywords:** aramid fiber composite, friction reduction, wear, performance of mixed rubber

## Abstract

Studies show that the long-term operation of a rubber mixer results in wear at the end face of the mixer. End face wear increases the gap between the mixing chamber and the end face, resulting in leakage and a reduction in the mixing performance, affecting the final product’s quality. Therefore, it is essential to investigate the wear of the metal face during the mixing process. The present study added aramid fibers to a rubber compound using a mechanical blender to obtain a composite material. Then, the influence of the aramid fibers on the metal friction and wear of the end face of the mixer was analyzed. This article introduces the concept of the wear ratio and explores the friction and wear of metals from the perspective of formulation technology for the first time. With the addition of aramid fibers, the proportion of abrasive wear of rubber on metal decreased, and the proportion of corrosive wear increased during the mixing process; however, when the addition of aramid fibers exceeded 3 phr, the balance of abrasive wear of rubber on metal increased and the proportion of corrosive wear decreased. It was found that aramid fibers have the property of friction reduction, which reduces the wear of the rubber blend on the metal. When the amount of aramid fibers added was 3 phr, the amount of abrasion of the rubber compound on the metal was the lowest.

## 1. Introduction

The addition of short fibers to rubber can improve some of its properties, as well as the processing performance [1,2,3]. Since the 1970s, people have conducted a lot of research on different short fibers, such as cellulose fibers, glass fibers, nylon fibers, polyester fibers, carbon fibers, and aramid fiber-reinforced skeleton materials. The improvement in rubber performance by different staple fibers also varies. Aramid fibers have high strength, a high modulus, high-temperature resistance, and excellent cutting and chemical corrosion [4]. Cutting aramid fibers into short fibers of specific gauge lengths and adding them to hybrid rubber to prepare hybrid rubber/fiber composites can improve the tearing properties and wear resistance and reduce the rolling resistance of the composites [5]. Some companies have used aramid fibers to prepare tires with better performance than existing technologies.

Zhang Yong [6] studied the application of aramid staple fibers in mud-tire tread rubber. The results showed the following: compared with the rubber without aramid staple fibers, the coking time and good vulcanization time of the rubber with the addition of aramid staple fibers were slightly extended, and the vulcanization reversion time was shortened; the cutting and puncture resistance of the vulcanized rubber was significantly improved, the loss factor was reduced, and the shear temperature rise was decreased dramatically; the best performance was achieved when the amount of aramid staple fibers was 3 phr; and the durability performance of the finished tire increased with the increase in the amount of aramid staple fibers. 

Xiao Jianbin [7] studied the effect of the addition of aramid staple fibers on the properties of natural rubber/fiber composites. The results showed the following: under the same mixing process conditions, the performance of the rubber/fiber composites improved and then decreased as the amount of aramid staple fibers added increased; the mechanical properties and the abrasion performance were the best when 3 phr of aramid staple fibers was added; the addition of aramid staple fibers reduced the thermal conductivity of the rubber; and the addition of aramid staple fibers improved the anti-slip performance and rolling resistance of the tread using rubber/fiber composites. The effects of the fiber length and fiber mass fraction on the transverse and longitudinal tensile properties of AFRC have also been investigated. Results have shown that the transverse and longitudinal tensile strengths of AFRC increase with the increase in the fiber mass fraction, and the longitudinal tensile strength is generally higher than the transverse tensile strength; the overall performance of AFRC is better when the fiber mass fraction is 4%, and the length is 3 mm.

Based on the secondary development of Python-ABAQUS, Liu Xia [8] generated a two-dimensional representative volume element of random aramid fiber-reinforced rubber composites considering the non-ideal interface between aramid fibers and the rubber matrix. Liu Xia applied periodic boundary conditions to the numerical model of aramid fiber-reinforced rubber composite (AFRC) for simulation analysis, combining this with uniaxial tensile experiments to study the effect of the fiber volume fraction on the tensile properties of AFRC. Liu Xia used the cohesion model to describe the mechanical behavior of the interface and analyzed the impact of interface properties on the axial tensile properties of AFRC. The results showed that, in the weak interfacial stiffness region, the effective elastic modulus of AFRC decreased with the increase in the fiber volume fraction. In the vital interface region, the effective elastic modulus of AFRC increased with the increase in the fiber volume fraction. The macroscopic effective elastic modulus measured in AFRC with different fiber contents can predict the cohesive force model parameters of the non-ideal interface through the stiffness modulus curve.

Liu Long [9] used a silane coupling agent for surface grafting modification of plain-woven Arlene fibers. He studied the effect of surface modification on the interlayer bonding properties and impact resistance of Arlene fiber/rubber matrix composites. FT-IR was used to analyze the surface of the modified aramid fibers, the modification principle was studied, and SEM was used to analyze the surface microstructure of the modified aramid fibers. The results showed that the interlaminar binding force and the peak impact load of the rubber matrix composite among threads were increased by 42% and 33.06%, respectively, after the surface modification. It was also shown that the improvement of the fiber surface had a positive effect on the low-speed impact resistance, which provides a new idea for the progress of the impact resistance of ARPA fiber/rubber matrix composites, and is of great significance for the application of composite materials in a complex environment.

Pang Li [10] studied the effect of modified aramid staple fibers of different lengths on the vulcanization characteristics, mechanical properties, and DIN friction properties of carbon nanotube-reinforced NR/BR composites, analyzing the friction surface using a stereo microscope. The results showed that when 1 mm aramid fibers were added under the same conditions, the tensile strength and elongation at break of the compound were better and the abrasion was higher; when 3 mm aramid fibers were added, the combination had 100% tensile stress, better tear strength, and lower wear.

As the rubber mixing equipment is continuously used for production in the factory, the internal mixer has a long working time and a high working intensity, which will inevitably bring about the wear of the end face of the internal mixer. The end face is an integral part of the closed mixing chamber, and the wear of the end face will cause the gap between the mixing room and the end face to increase. The increase in the opening leads to the phenomenon of material leakage [11,12,13,14]. With the intensification of the leakage, the gap between the end face of the mixer and the mixing chamber will further increase, thus forming negative feedback. This phenomenon not only makes the formula ratio inaccurate but also leads to a decrease in the mixing effect and a reduction in the performance of the compounded rubber. This paper used aramid fibers as a filler, and aramid fiber/rubber composites were prepared by mechanical blending. The influence of different ratios of aramid fiber/rubber composites on the friction and wear of the end face of the internal mixer was analyzed.

## 2. Experiment

### 2.1. Chemical Composition

The chemical composition of the prepared samples is shown in Table 1.

Highly dispersible RFL pre-impregnated aramid staple fibers were used (1.5D length 3 mm, Qingdao-Sanxiong Fiber Technology Co., Qingdao, China).

### 2.2. Sample Preparation

#### 2.2.1. Natural Rubber (NR) Plasticizing

Natural rubber is a hard-to-mix substance in the mixer. To facilitate the mixing process, it was necessary to open the mixer for plasticizing. Before plasticizing, the mixer was washed to reduce the influence of roller impurities on the rubber performance [15]. Natural rubber (NR) was plasticized by an opening mill (BL-6157, Baolun Precision Testing Instrument Co., Dongguan, China). During the plasticizing process, the cooling water temperature was set to 40–50 °C. Finally, the steps of the process were as follows: rubber breaking 4 times (roller distance 4 mm)→10 times thin (roller distance 0.5 mm)→2 times (roller distance 3 mm) [16,17,18,19,20,21]^.^

#### 2.2.2. Preparation of the Mixed Rubber

The mixing process was carried out in a dense mixer (XSM-500, Shanghai Kechuang Rubber, and Plastic Machinery Equipment Co., Shanghai, China); the rotor speed and cooling water temperature were set to 70 r/min and 40 °C, respectively. Moreover, the filling coefficient and upper-plug pressure were set to 0.65 and 0.6 MPa, respectively. Then, the plasticizer was cut into thin strips for easy feeding. The preparation process of the mixed rubber is shown in Table 2.

The glue was plasticized in the open mixer after passing three times, and the triangle bag was rolled five times. Finally, the roller distance of the mixer was set to 10 mm to obtain a smooth surface on the sample.

### 2.3. Performance Test

#### 2.3.1. Rubber Processing Performance

**Figure 1:**
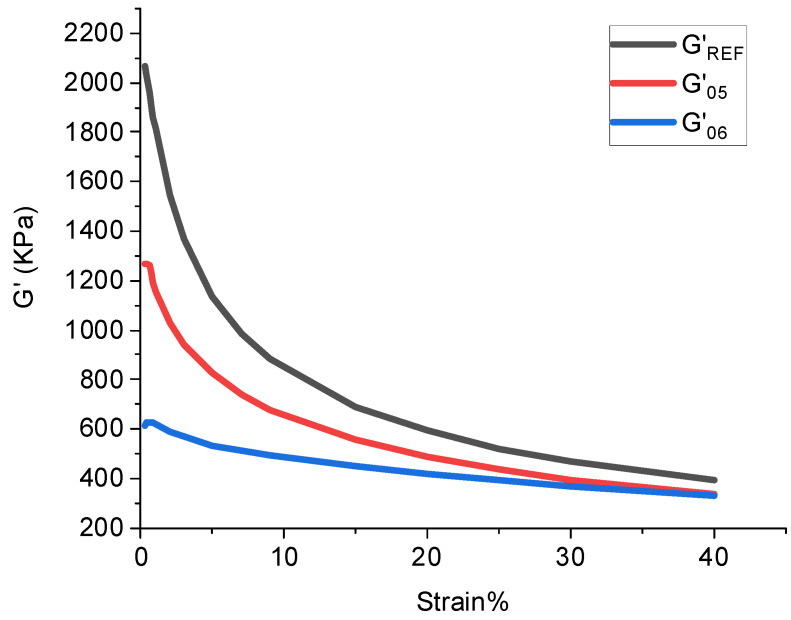
Distribution of silanization reaction degree.

The rubber processing performance was tested using the RPA 2000 (Rubber processing performance analyzer, Alpha Company, USA) test. During the experiment, the scanning frequency, scanning strain range, and temperature were set to 0.01 Hz, 0.28–40%, and 60 °C, respectively [22,23,24,25,26,27,28,29]. The dynamic modulus G’ curve with strain was obtained. Moreover, the following indicators were calculated during the experiment:

(1) Payne effect: The Payne effect refers to the phenomenon that the dynamic modulus of a filled rubber decreases sharply with increasing strain. This effect reflects the distribution of the cross-linking network inside the rubber. Generally, the higher the Payne effect, the denser the cross-linking network inside the rubber, and the worse the filler dispersity [30,31,32,33,34,35].

(2) Silanization reaction index: G’ and strain obtained by the silica multi-path stable cooling method were calculated to obtain the silanization reaction index. The calculation method of the silanization reaction index is shown in Table 3. The calculation method of the silanization reaction is shown in Figure 1.

$$X=\frac{\mathrm{Area}\;\mathrm{of}\;\mathrm{silylation}\;\mathrm{zoon}}{\mathrm{Area}\;\mathrm{of}\;\mathrm{the}\;\mathrm{largest}\;\mathrm{silylation}\;\mathrm{region}}=\frac{\int {G’}_{REF}(05)-\int {G’}_{S}(05)}{\int {G’}_{REF}(05)-\int {G’}_{S}(06)}$$

#### 2.3.2. CSM Friction and Wear Test

**Figure 2:**
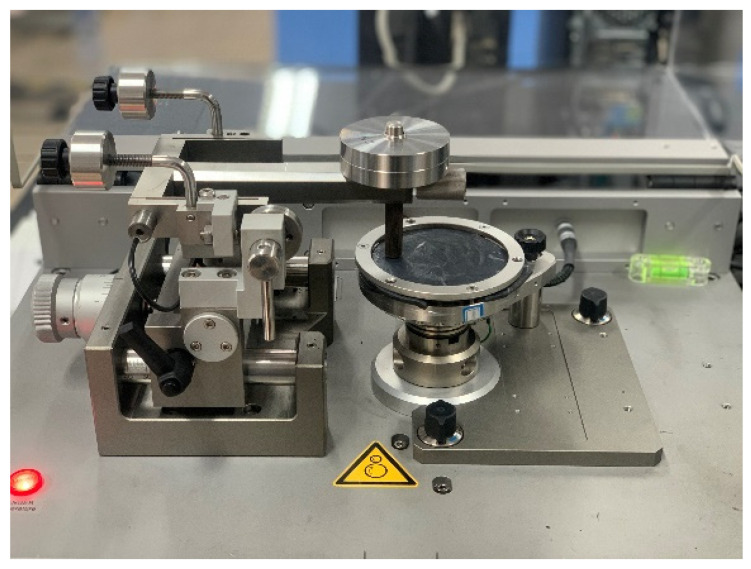
Configuration of the CSM wear experiment.

Circular rubber samples with a diameter of 100 mm were prepared by cutting the rubber sample with a smooth surface with the CSM (CSM- Friction and wear testing machine, Tribometer company, Switzerland) mold. After calibrating the CSM friction and wear tester, the parameters of the CSM were set according to the mixing process parameters. The pressure, speed, and friction time were set to 5 N, 70 r/min, and 30 min, respectively. To accurately simulate the mixing situation of the mixer, a metal grinding head was used in the CSM test, which was consistent with the end face of the mixer. Studies have shown that metal wear increases significantly at high temperatures in the mixing process [36,37,38,39,40,41,42,43,44,45]. Accordingly, the CSM temperature was set at 150 °C to observe the wear effect better. The configuration of the CSM is presented in Figure 2.

#### 2.3.3. Three-Dimensional Morphology of Metal

A 3D scanner (LEXT OLS5000, Olympus, Japan) was used to analyze the surface of the test sample after scanning, and the variation in the morphology and volume of the sample before and after wear was obtained.

#### 2.3.4. Dispersion Test

The vulcanized rubber was cut out into a new section, and the cut section was tested using the dispersibility tester (Carbon black dispersing meter, Alpha Company, Hudson, OH, USA). The dispersibility value was obtained according to the ASTM D7723 standard.

## 3. Silanization Reaction Mechanism

**Figure 3:**
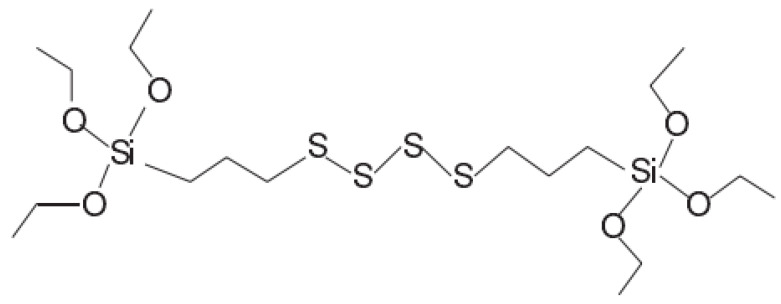
Chemical composition and molecular structure of TESPT.

In all experiments, TESPT was used as the silane coupling agent. Figure 3 illustrates the chemical composition and molecular structure of TESPT.

**Figure 4:**
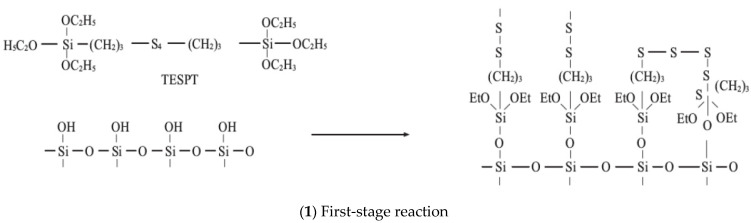

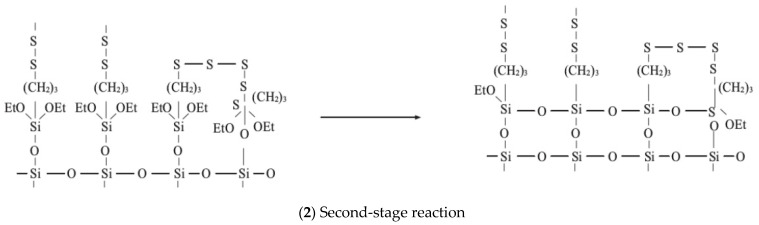
The silanization reaction.

The silanization reaction between TESPT and silica can be divided into two steps, as shown in Figure 4.

(1) First-stage reaction: In the first stage, the alkoxy group of TESPT dealcoholizes with the Si-OH group of silica, and the Si-OR group in TESPT hydrolyzes and forms a free Si-OH group. Accordingly, the dehydration condensation of the -Oh group starts on the silica surface.

(2) Second-stage reaction: The condensation reaction between adjacent silane coupling agents chemically bound to the surface of white carbon black.

In the second mixing stage, the mixing chamber temperature increases, and the mixer is closed. The high-temperature ethanol–water mixed steam cannot overflow in the mixer under the high-temperature environment of the mixing chamber. Therefore, the corrosion wear induced by the high-temperature steam should be considered in the friction and wear of the end face. However, the mixer is a massive device that can hardly be disassembled. Subsequently, the ethanol–water vapor produced in the mixing process cannot be measured directly. The silanization reaction index is commonly used to characterize the silanization reaction degree in the rubber industry. To accurately simulate the mixing situation of the mixer during the friction test, high-temperature ethanol–water mixed vapor was sprayed on the mixing rubber and metal surface according to the silanization reaction.

## 4. Experimental Results

### 4.1. Analysis of Rubber Processing

#### 4.1.1. Payne Effect

**Figure 5:**
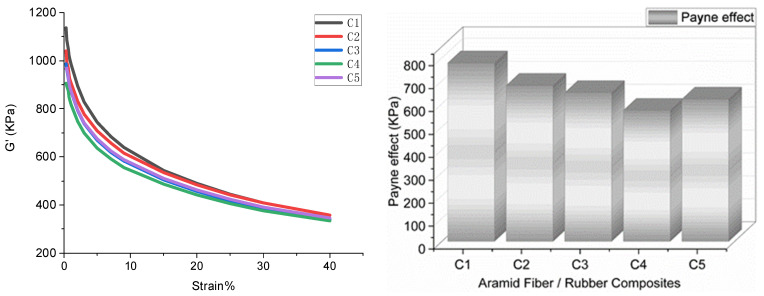
Payne effect.

**Figure 6:**
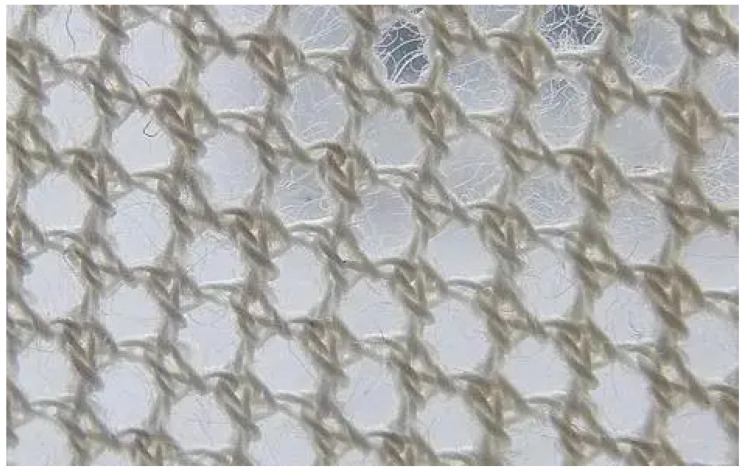
Microstructure of the aramid fiber (20× magnification).

Figure 5 shows the Payne effect of five types of blends. This experiment used high-dispersion RFL pre-impregnated aramid staple fibers (1.5D length 3 mm). The aramid fiber is a new high-tech synthetic fiber with super high strength, a high modulus, high-temperature resistance, acid resistance, and alkali resistance. It is lightweight and has other excellent properties: it is 5–6 times stronger than steel wire, its modulus is 2–3 times that of steel wire or glass fiber, its toughness is 2 times that of steel wire, and its weight is only about 1/5 of that of steel wire. The microstructure of the aramid fiber is shown in Figure 6. The aramid fiber has a vast surface area, and its surface can adsorb SiO_2_ particles, which promotes the dispersion of SiO_2_ particles.

No aramid fibers were added to the C1 formulation. There was no adsorption of aramid fibers on SiO_2_ particles to further promote the dispersion of SiO_2_ particles, so the silanization reaction was low, and the Payne effect was significant.

As the addition of aramid fibers continued, the surface area of aramid fibers within the compound increased and the adsorbed SiO_2_ particles increased. Therefore, the Payne effect of the C3 and C4 formulations of the compounding rubber continued to decrease. When the amount of aramid fibers added was 3 phr, the Payne effect of the compound was the smallest, and the SiO_2_ particle dispersion was the best at this time. As the addition of aramid fibers continued, agglomeration between the aramid fibers occurred, reducing the aramid fibers’ surface area within the compound. Therefore, the dispersion of SiO_2_ particles combined with 4 phr of aramid fibers decreased, and the Payne effect increased.

#### 4.1.2. Rubber Dispersion Test

**Figure 7:**
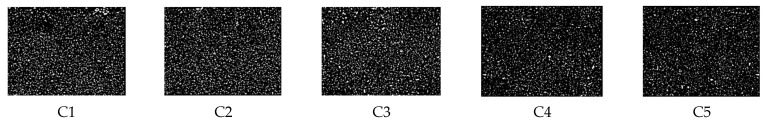
Dispersion image.

Figure 7 shows the dispersion image of the rubber, and the dispersion value is presented in Table 4. The rubber dispersion test mainly focuses on SiO_2_ particle aggregates, so rubber dispersion corresponds to the Payne effect.

### 4.2. Silanization Reaction Index

**Figure 8:**
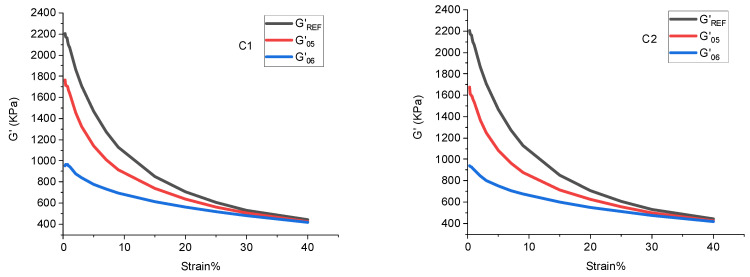

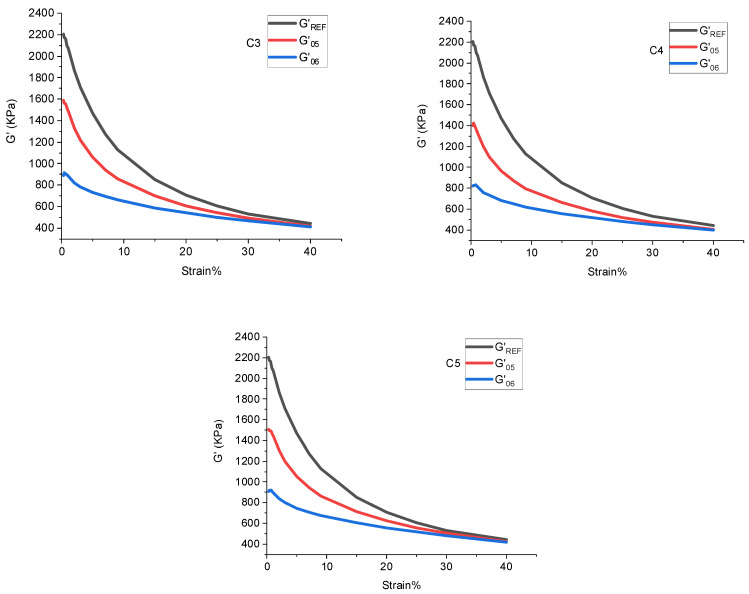
Distributions of the silanization reaction degree.

The silanization reaction degree of the rubber is shown in Figure 8 and Table 5. The silanization reaction degree reflects the compound’s dispersion of SiO_2_ particles. It was found that when the amount of aramid fibers in the combination increased gradually, more SiO_2_ particles could be absorbed, and the dispersion of SiO_2_ particles increased. Accordingly, the silanization reaction degree gradually increased. When the amount of aramid fibers in the compound exceeded 3 phr, the agglomeration phenomenon occurred, decreasing the aramid fibers’ absorbable surface area. Meanwhile, the aggregate of aramid fiber particles had a large volume and was dispersed in the mixing glue, which hindered the dispersion of SiO_2_ particles. Therefore, the degree of the silanization reaction of the compound with 4 phr of aramid fibers decreased.

In the field of the rubber industry, the silanization reaction index is commonly used to characterize the product qualitatively. High-temperature ethanol–water mixed vapor was sprayed on the mixing rubber and the metal surface to simulate the mixing process in the friction test. The spray flow rate was in proportion to the degree of the silanization reaction. Considering the ratio of the silanization reaction index in the present study, 150 °C ethanol–water mixed vapor was sprayed at 1:1.13:1.21:1.39:1.22.

### 4.3. CSM Friction and Wear Test

#### 4.3.1. Average Friction Coefficient

**Figure 9:**
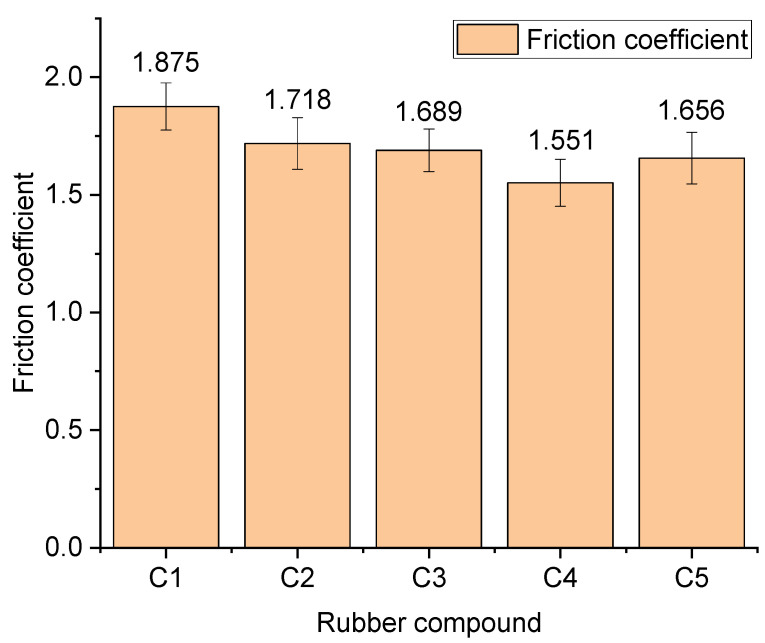
Average friction coefficient.

Figure 9 shows the average friction coefficient measured in the CSM experiment.

Aramid fibers have a friction reduction property, mainly from their unique structure. As the amount of aramid fibers in the compound increased, the friction coefficient of the compound on the metal gradually decreased. Meanwhile, the absorbed SiO_2_ particles on the surface of the aramid fiber particles promoted the dispersion of SiO_2_ particles. Accordingly, the distribution of SiO_2_ particles improved, and the accumulation of SiO_2_ particles in the compound decreased, thereby reducing the compound friction on the metal. When the amount of aramid fibers in the combination exceeded 3 phr, the aramid fiber particles agglomerated, decreasing the absorbable surface area of the aramid fiber particles. The greater the accumulation, the lower the antifriction property of the aramid fibers, which increases the average friction coefficient of the mixing glue on the metal.

#### 4.3.2. Three-Dimensional Morphology of the Metal Grinding Head

**Figure 10:**
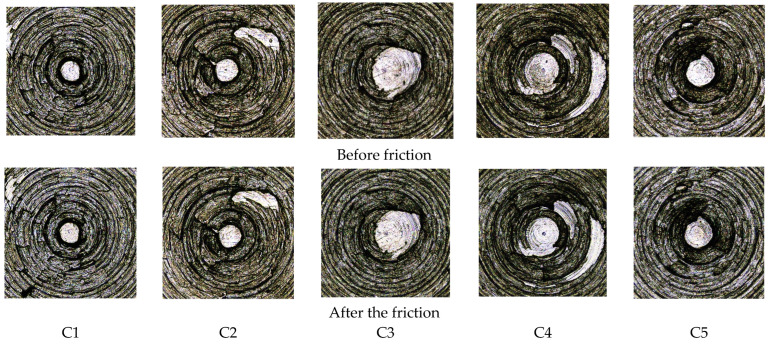
Surface morphology of the metal grinding head before and after friction.

**Figure 11:**
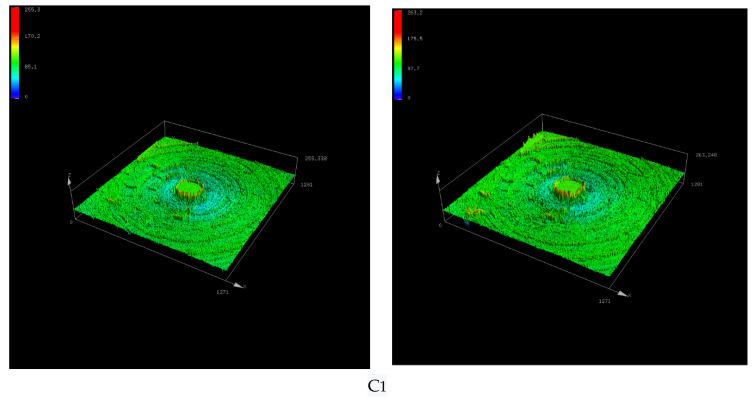

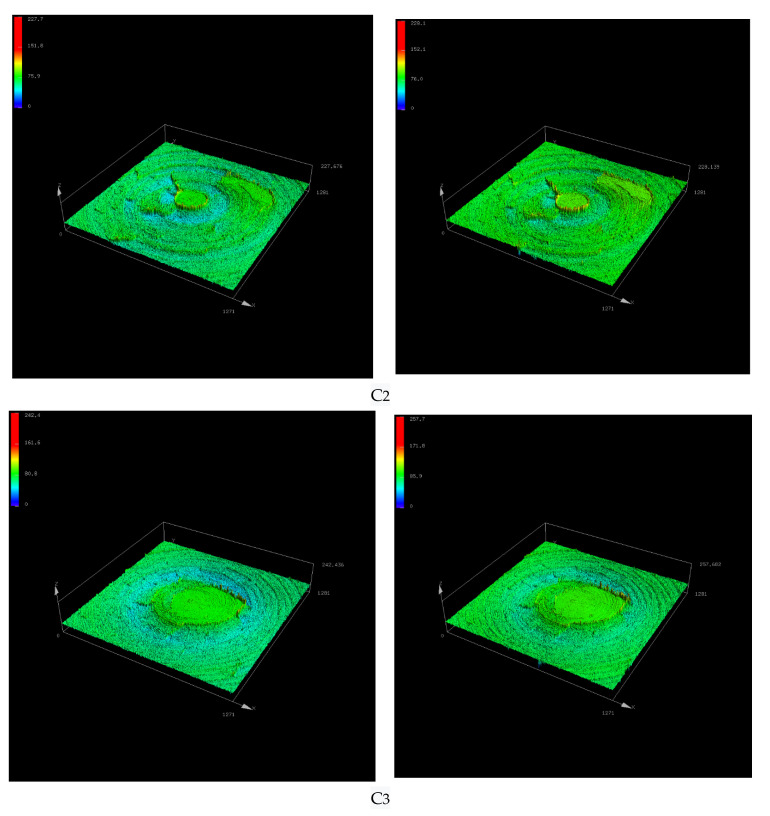

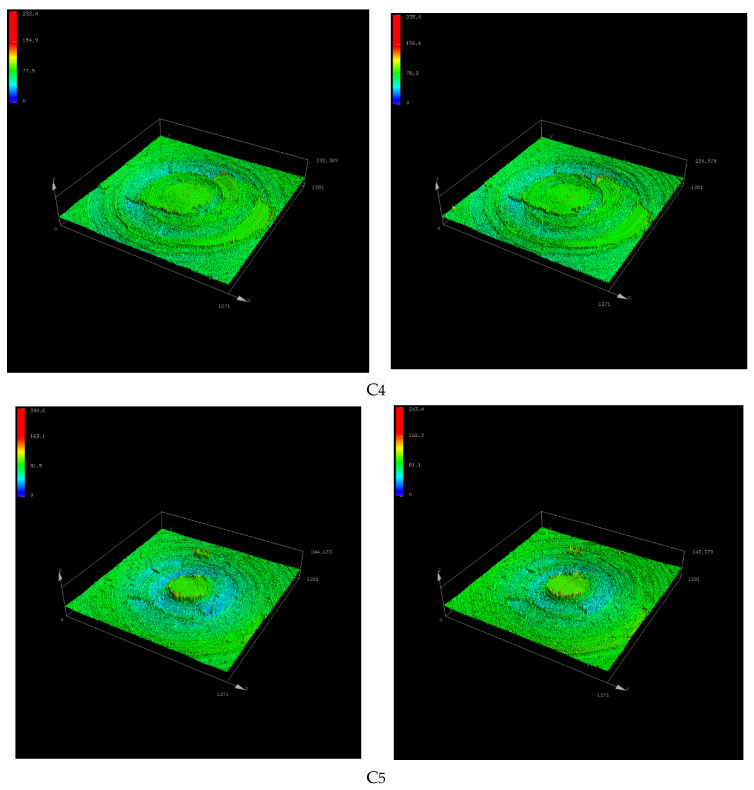
Three-dimensional morphology of the metal surface before and after friction.

Figure 10 shows the surface morphology of the metal grinding head with ten times magnification, and Figure 11 shows the three-dimensional morphology of the metal grinding head surface with ten times magnification. From Figure 10 and Figure 11, it can be seen that the three-dimensional shape of the metal surface of the C1 formulation had more areas of color deepening after rubbing, which indicates that the volume loss of the metal grinding head was greater. The C2, C3, and C4 formulations of the compounding rubber before and after rubbing the three-dimensional shape of the metal surface had a minor color change, indicating that the metal’s volume loss was reduced. The color-deepening area increased for the C5 formulation, which suggests that the abrasion of the metal increased for the C5 formulation relative to the C4 formulation.

#### 4.3.3. Height Profiles before and after Friction

**Figure 12:**
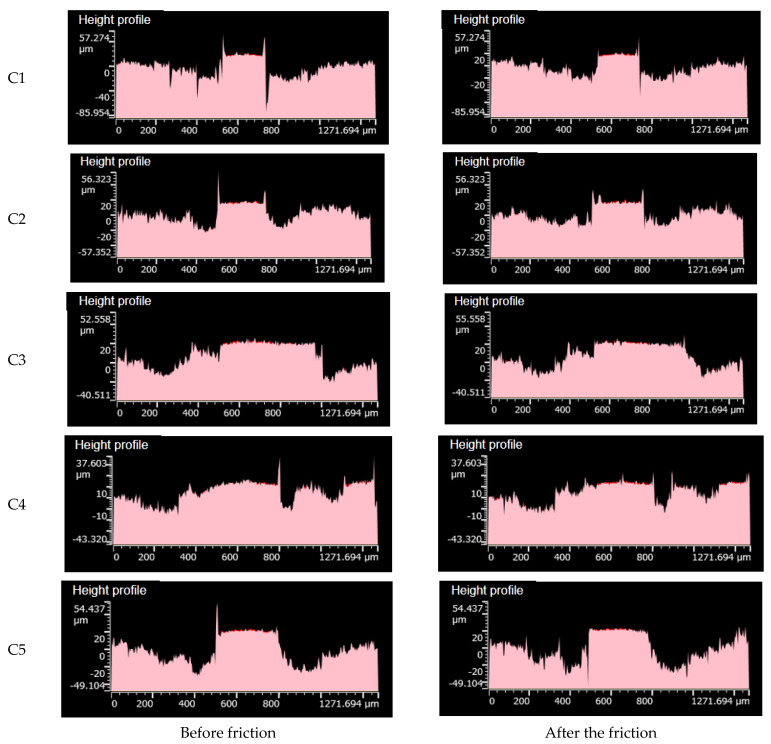
Height profiles before and after friction.

Figure 12 shows the height profile of the metal grinding head before and after friction. It was observed that the metal surface height profile subjected to the C1 rubber composition changed significantly. The surface height profile after friction was flattened, and more spikes appeared. Variations in the metal surface height profile of the C2 and C3 pieces were less than those of C1, and the lowest variations were achieved with the C4 arrangement. This may be attributed to the absorption of SiO_2_ particles on the surface of aramid fiber particles, which promoted the dispersion of SiO_2_ particles and reduced the SiO_2_ agglomeration. This is because agglomerated SiO_2_ particles have high hardness and a strong character, intensifying metal wear.

Meanwhile, as the amount of aramid fibers in the rubber compound increased, friction further reduced, and the metal wear weakened. When the amount of aramid fibers in the combination exceeded 3 phr, the aramid fiber particles agglomerated, decreasing the adsorbable surface area of the aramid fiber particles. The aggregated aramid fiber particles were relatively large and were dispersed in the mixing glue, hindering the dispersion of SiO_2_ particles and increasing the number of SiO_2_ particle aggregates. Therefore, the compound with 4 phr of aramid fibers had high metal wear.

#### 4.3.4. Change in Metal Volume before and after Friction

**Figure 13:**
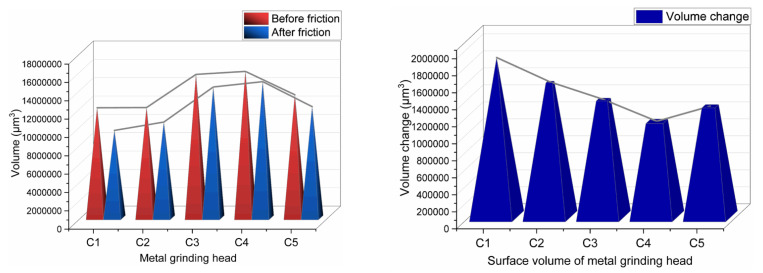
Change in the metal volume before and after friction.

The change in the metal volume before and after friction is shown in Figure 13.

Figure 13 indicates that as the amount of aramid fibers in the rubber compound increased, metal wear decreased first and then increased. This phenomenon may be attributed to the unique structure of aramid fibers. When the amount of aramid fibers is less than 3 phr, SiO_2_ adheres to the surface of aramid fiber particles, thereby promoting the dispersion of SiO_2_ particles and reducing the aggregation of SiO_2_ particles. It is worth noting that these aggregated particles have a high hardness and sharp surface, which is the most critical parameter of metal wear. Accordingly, the lower the aggregation of SiO_2_ particles, the lower the metal wear.

On the other hand, as the amount of aramid fibers in the compound increased, the substance friction and wear reduced. When the aramid fiber content exceeded 3 phr, the aramid fiber particles aggregated, decreasing the aramid fibers’ adsorbable surface area. Since aggregated aramid fiber particles are relatively large, they were dispersed in the mixing glue, hindering the dispersion of SiO_2_ particles and increasing the number of aggregated SiO_2_ particles. Accordingly, the compound with 4 phr of aramid fibers had relatively high metal wear.

#### 4.3.5. Proportion of Corrosion Wear to Abrasive Wear

**Figure 14:**
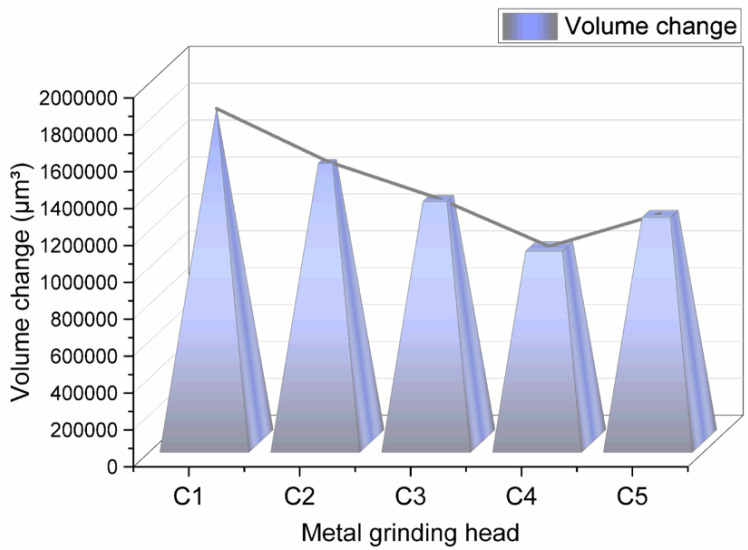
Metal wear volume without spraying the ethanol–water mixture.

In this part, experiments were carried out while no ethanol–water vapor was sprayed. Therefore, the measured wear quantity was abrasive wear. The variation in the metal volume before and after the friction experiment is shown in Figure 14.

**Figure 15:**
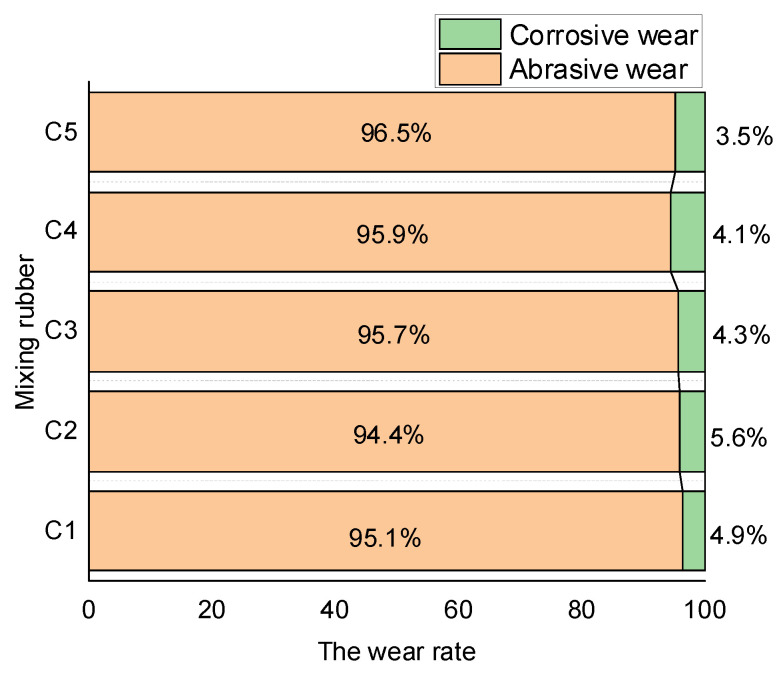
The ratio of the two wear mechanisms.

The metal’s abrasive and corrosion wear could be calculated separately by comparing the measured wear volumes in the experiment with and without spewing the ethanol–water mixed steam. To ensure accuracy in the investigation, all tests were repeated six times, and then the obtained values were averaged. The proportions of the two wear mechanisms are shown in Figure 15.

Corrosion wear mainly originated from the high-temperature ethanol–water vapor. On the other hand, the yield of the ethanol–water moisture was directly related to the degree of the silanization reaction. By adding aramid fibers to the rubber compound, the total surface area of the aramid fiber particles increased so that more SiO_2_ particles could be adsorbed, which promoted the dispersion of SiO_2_ particles. Accordingly, as the amount of aramid fibers in the rubber compound increased, the distribution of SiO_2_ particles improved, and the degree of the silanization reaction gradually increased. Moreover, the amount of high-temperature ethanol–water vapor increased, as well as the proportion of corrosion and wear additions. When the aramid fibers in the rubber compound exceeded 3 phr, the aramid fiber particles agglomerated. This phenomenon decreased the adsorbable surface area of the aramid fiber particles, thereby reducing the dispersion of SiO_2_ particles. Consequently, the silanization reaction in the compound with 5 phr of aramid fibers reduced, the amount of high-temperature ethanol–water vapor decreased, and the proportion of corrosion to wear dropped.

#### 4.3.6. Changes in Metal Surface Roughness before and after Friction

**Figure 16:**
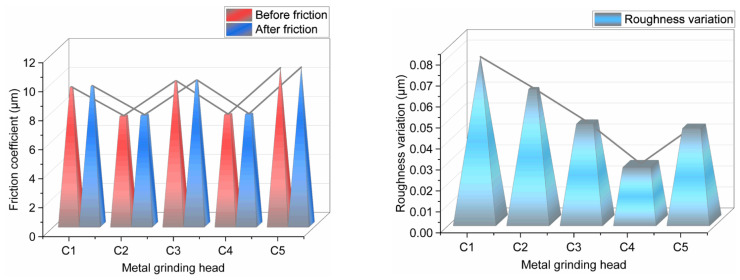
Metal surface roughness before and after friction.

Figure 16 illustrates the variation in the metal surface roughness before and after the friction of several mixing adhesives.

Figure 16 indicates that the roughness of the metal surface increased after friction. Silica has a high hardness and a strong character, and the accumulation of SiO_2_ particles is the most critical factor affecting the metal surface roughness. When the amount of aramid fibers in the rubber compound was less than 3 phr, SiO_2_ particles were attached to the surface of the aramid fiber particles, thereby promoting the dispersion of SiO_2_ particles as well as reducing their accumulation. Meanwhile, as the amount of aramid fibers in the rubber compound increased, the friction reduction of the particles intensified, and metal wear reduced. Therefore, the metal surface roughness decreased continuously. When the aramid fiber content of the rubber compound exceeded 3 phr, aramid fiber particles aggregated, thereby reducing the adsorbable surface area of the aramid fiber particles, reducing the dispersion of SiO_2_ particles, and increasing the aggregation of SiO_2_ particles. Therefore, after mixing the glue with 4 phr of aramid fibers, the roughness of the metal surface increased.

## 5. Conclusions

In this experiment, the frictional wear of rubber on metal during the mixing process was studied by applying the silylation reaction mechanism and the frictional wear of metal from the perspective of the formulation process for the first time. With the addition of aramid fibers, the average friction coefficient and the amount of change in metal roughness between the rubber and metal during the mixing process gradually decreased and were the lowest when the addition amount of aramid fibers was 3 phr. When the addition of aramid fibers exceeded 3 phr, the rubber and metal friction coefficient and roughness increased gradually. With the addition of aramid fibers, the proportion of abrasive wear of rubber on metal in the mixing process decreased, and the balance of corrosion wear increased. However, when the addition of aramid fibers exceeded 3 phr, the proportion of abrasive wear of rubber on metal increased, and the ratio of corrosion wear decreased. When the amount of aramid fiber addition was 3 phr, the abrasive wear of rubber on metal was the smallest.

## Figures and Tables

**Table 1:** Chemical composition of the prepared samples.

Raw Material	C1	C2	C3	C4	C5
BR9000	25.5	25.5	25.5	25.5	25.5
RC2557S	81.81	81.81	81.81	81.81	81.81
TSR20	15	15	15	15	15
N234	10	10	10	10	10
Silica115MP	45	45	45	45	45
TESPT	6	6	6	6	6
ZnO	2	2	2	2	2
SAD	2	2	2	2	2
4020	2	2	2	2	2
DPG	0.8	0.8	0.8	0.8	0.8
Aramid fiber	0	1	2	3	4
S	1.3	1.3	1.3	1.3	1.3
CZ	1.8	1.8	1.8	1.8	1.8

**Table 2:** The preparation process of the mixed rubber.

1.6 L Mixer, 70 rpm, 75% FF		
Time	T (°C)	Ingredients
Master batch		
0:00	40	Polymers
0:40		Chemical, 1/2 Silica115 MP
1:10		1/2 Silica115 MP
2:30	120	Sweep
4:00	135	Sweep, Sampling
5:00	145	Discharge

**Table 3:** Stages of the RPA method to measure the degree of the silanization reaction.

Stage	Frequency/Hz	T/℃	Time/min	Strain	Test Items
1	0.1	60	5	0.28%	-
2	1	60	-	0.28–40%	*G’* (02)
3	1	60	-	0.28–40%	*G’* (03)
4	0.1	60/160/160	0/2.5/5	0.28%	-
5	1	60	-	0.28–40%	*G’* (05)
6	1	60	-	0.28–40%	*G’* (06)

**Table 4:** Dispersion values.

Rubber Compound	C1	C2	C3	C4	C5
Dispersion	5.14	5.57	5.97	6.68	6.39

**Table 5:** Silanization reaction index of five blends.

Rubber Compounds	C1	C2	C3	C4	C5
Silanization reaction index	0.463	0.525	0.559	0.642	0.563

## Data Availability

The data presented in this study are available on request from the corresponding author.
